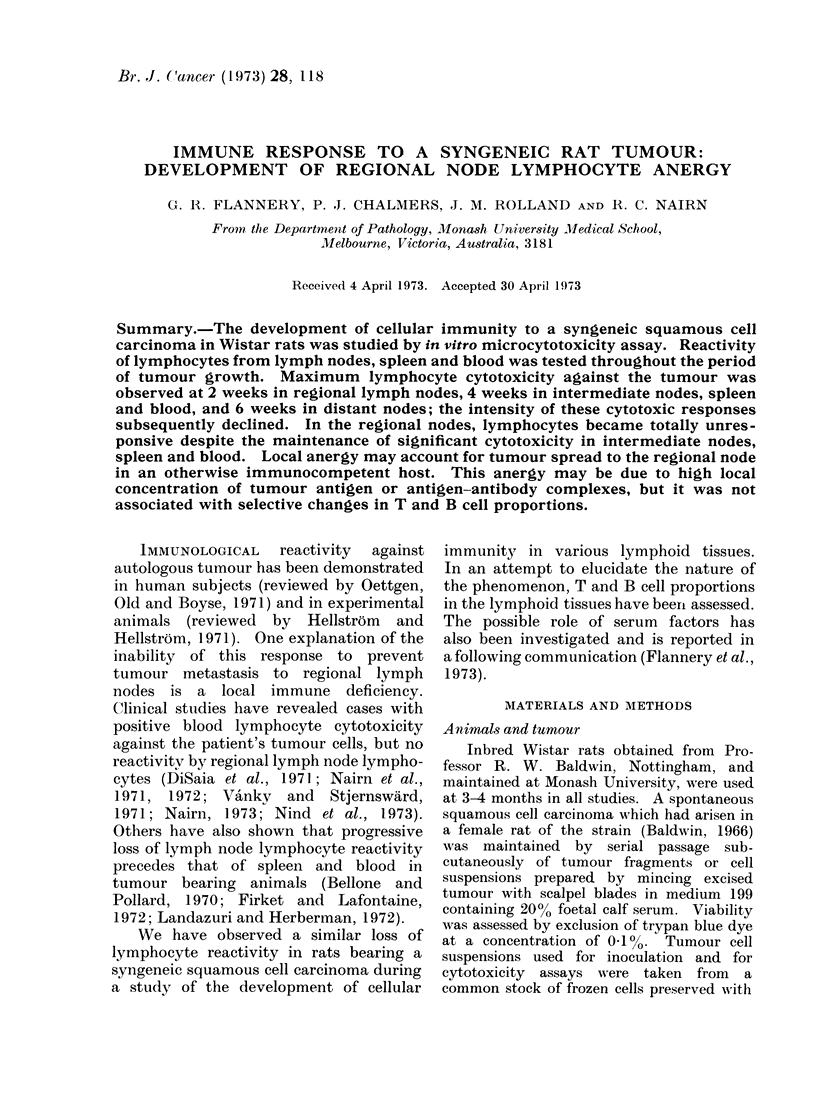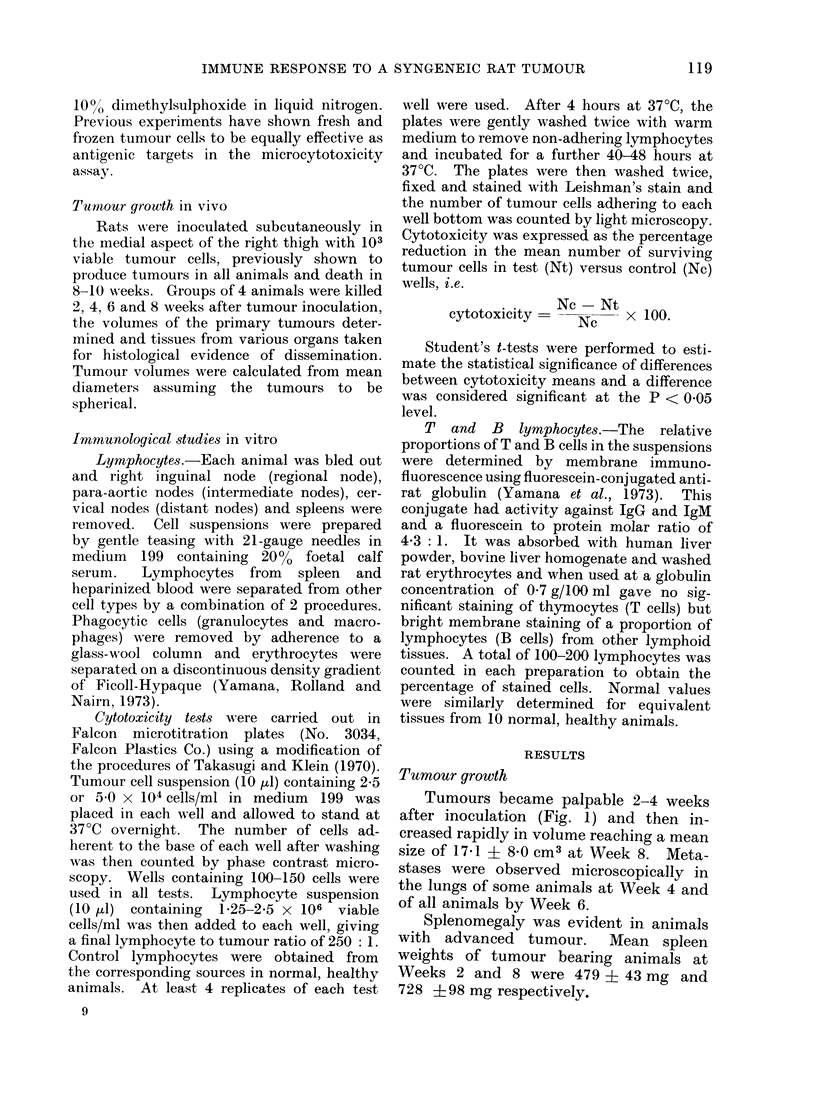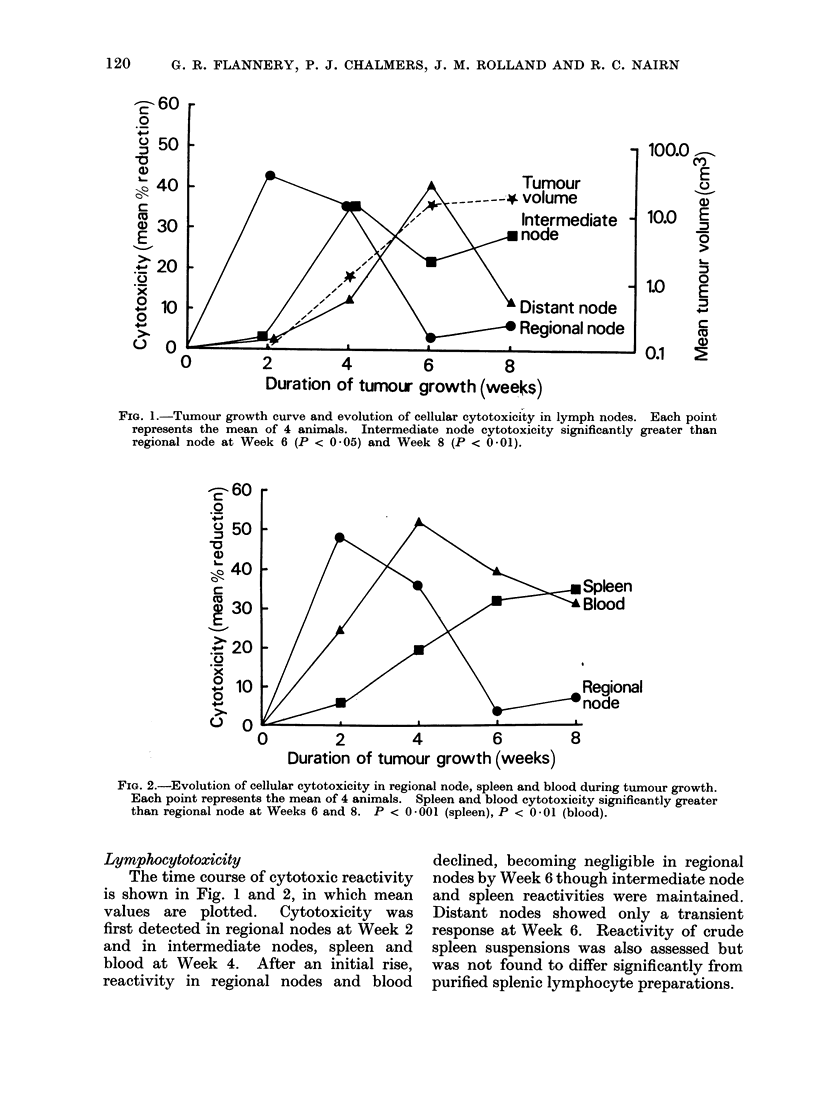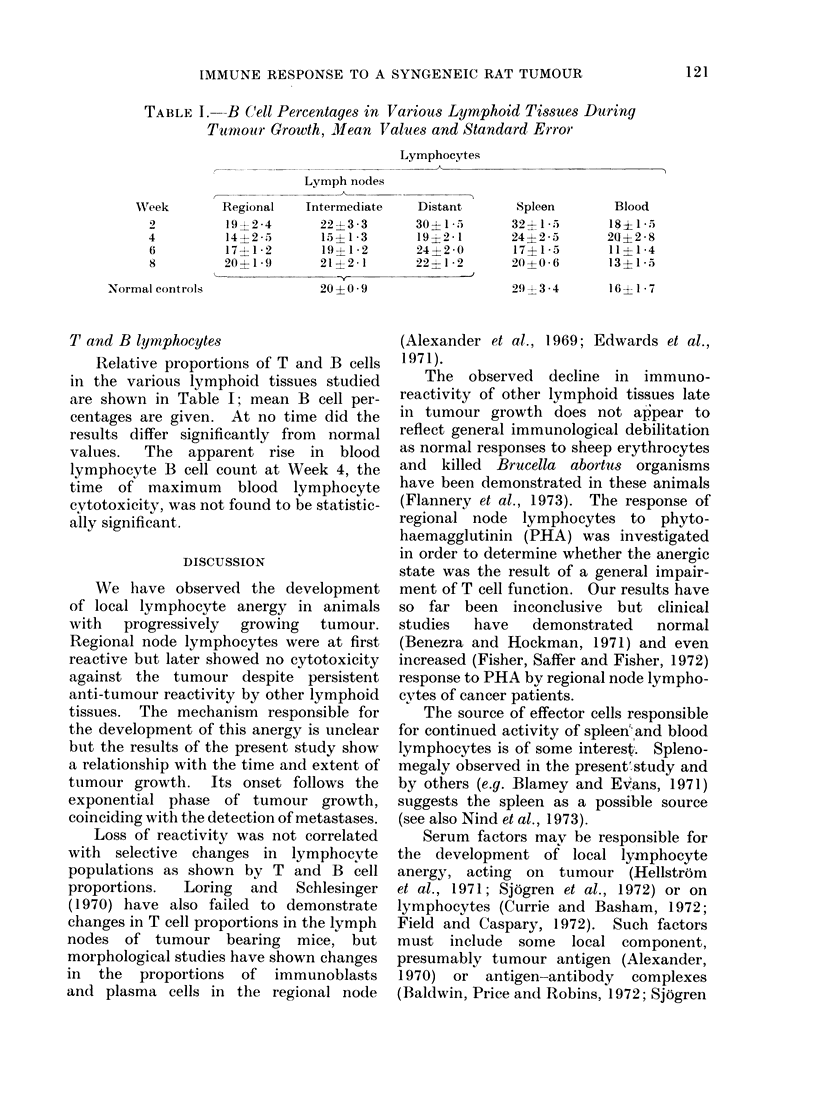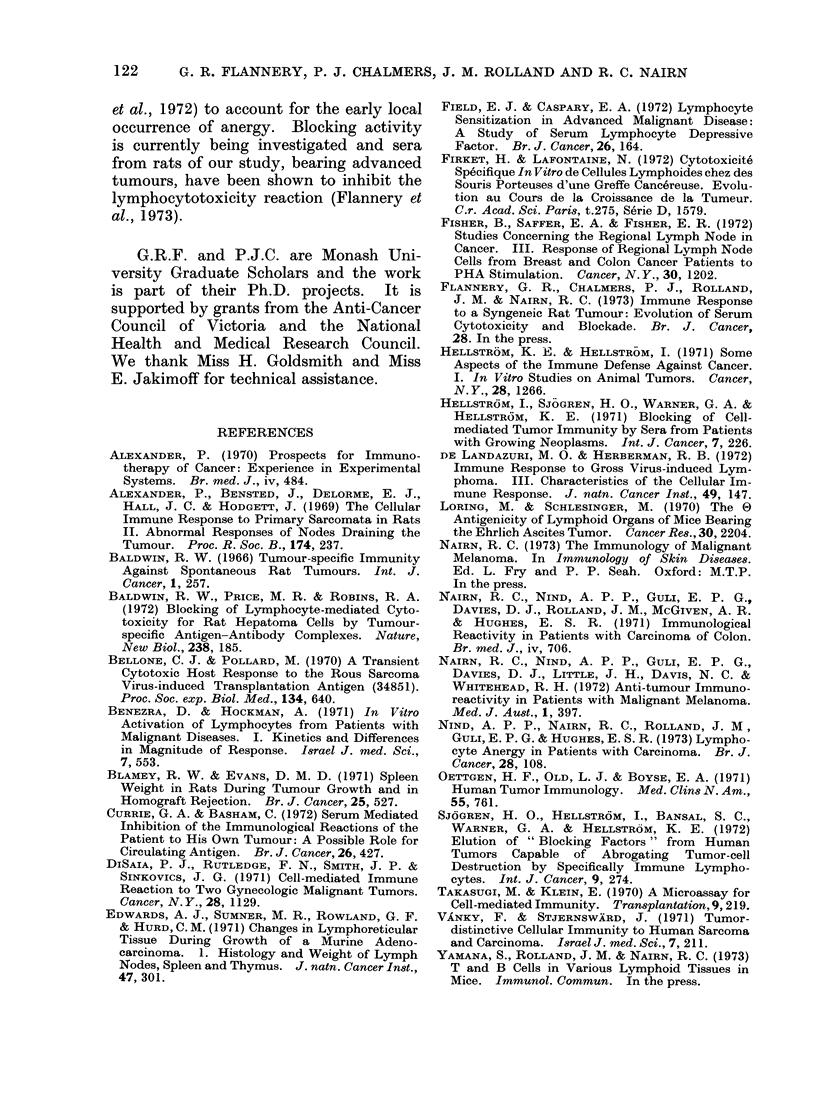# Immune Response to a Syngeneic Rat Tumour: Development of Regional Node Lymphocyte Anergy

**DOI:** 10.1038/bjc.1973.129

**Published:** 1973-08

**Authors:** G. R. Flannery, P. J. Chalmers, J. M. Rolland, R. C. Nairn

## Abstract

The development of cellular immunity to a syngeneic squamous cell carcinoma in Wistar rats was studied by *in vitro* microcytotoxicity assay. Reactivity of lymphocytes from lymph nodes, spleen and blood was tested throughout the period of tumour growth. Maximum lymphocyte cytotoxicity against the tumour was observed at 2 weeks in regional lymph nodes, 4 weeks in intermediate nodes, spleen and blood, and 6 weeks in distant nodes; the intensity of these cytotoxic responses subsequently declined. In the regional nodes, lymphocytes became totally unresponsive despite the maintenance of significant cytotoxicity in intermediate nodes, spleen and blood. Local anergy may account for tumour spread to the regional node in an otherwise immunocompetent host. This anergy may be due to high local concentration of tumour antigen or antigen-antibody complexes, but it was not associated with selective changes in T and B cell proportions.


Br. J. Cancer (1973) 28, 118

### IMMUNE RESPONSE TO A SYNGENEIC RAT TUMOUR: DEVELOPMENT OF REGIONAL NODE LYMPHOCYTE ANERGY

G. R. FLANNERY, P. J. CHALMERS, J. M. ROLLAND AND R. C. NAIRN

*From the Department of Pathology, Monash University Medical School, Melbourne, Victoria, Australia, 3181*

Received 4 April 1973. Accepted 30 April 1973

**Summary.—The development of cellular immunity to a syngeneic squamous cell carcinoma in Wistar rats was studied by *in vitro* microcytotoxicity assay. Reactivity of lymphocytes from lymph nodes, spleen and blood was tested throughout the period of tumour growth. Maximum lymphocyte cytotoxicity against the tumour was observed at 2 weeks in regional lymph nodes, 4 weeks in intermediate nodes, spleen and blood, and 6 weeks in distant nodes; the intensity of these cytotoxic responses subsequently declined. In the regional nodes, lymphocytes became totally unresponsive despite the maintenance of significant cytotoxicity in intermediate nodes, spleen and blood. Local anergy may account for tumour spread to the regional node in an otherwise immunocompetent host. This anergy may be due to high local concentration of tumour antigen or antigen–antibody complexes, but it was not associated with selective changes in T and B cell proportions.**

IMMUNOLOGICAL reactivity against autologous tumour has been demonstrated in human subjects (reviewed by Oettgen, Old and Boyse, 1971) and in experimental animals (reviewed by Hellström and Hellström, 1971). One explanation of the inability of this response to prevent tumour metastasis to regional lymph nodes is a local immune deficiency. Clinical studies have revealed cases with positive blood lymphocyte cytotoxicity against the patient's tumour cells, but no reactivity by regional lymph node lymphocytes (DiSaia *et al.*, 1971; Nairn *et al.*, 1971, 1972; Vánky and Stjernswärd, 1971; Nairn, 1973; Nind *et al.*, 1973). Others have also shown that progressive loss of lymph node lymphocyte reactivity precedes that of spleen and blood in tumour bearing animals (Bellone and Pollard, 1970; Firket and Lafontaine, 1972; Landazuri and Herberman, 1972).

We have observed a similar loss of lymphocyte reactivity in rats bearing a syngeneic squamous cell carcinoma during a study of the development of cellular immunity in various lymphoid tissues. In an attempt to elucidate the nature of the phenomenon, T and B cell proportions in the lymphoid tissues have been assessed. The possible role of serum factors has also been investigated and is reported in a following communication (Flannery *et al.*, 1973).

### MATERIALS AND METHODS

### *Animals and tumour*

Inbred Wistar rats obtained from Professor R. W. Baldwin, Nottingham, and maintained at Monash University, were used at 3–4 months in all studies. A spontaneous squamous cell carcinoma which had arisen in a female rat of the strain (Baldwin, 1966) was maintained by serial passage subcutaneously of tumour fragments or cell suspensions prepared by mincing excised tumour with scalpel blades in medium 199 containing 20% foetal calf serum. Viability was assessed by exclusion of trypan blue dye at a concentration of 0·1%. Tumour cell suspensions used for inoculation and for cytotoxicity assays were taken from a common stock of frozen cells preserved with

10% dimethylsulphoxide in liquid nitrogen. Previous experiments have shown fresh and frozen tumour cells to be equally effective as antigenic targets in the microcytotoxicity assay.

*Tumour growth* in vivo

Rats were inoculated subcutaneously in the medial aspect of the right thigh with $10^3$ viable tumour cells, previously shown to produce tumours in all animals and death in 8–10 weeks. Groups of 4 animals were killed 2, 4, 6 and 8 weeks after tumour inoculation, the volumes of the primary tumours determined and tissues from various organs taken for histological evidence of dissemination. Tumour volumes were calculated from mean diameters assuming the tumours to be spherical.

*Immunological studies* in vitro

*Lymphocytes.*—Each animal was bled out and right inguinal node (regional node), para-aortic nodes (intermediate nodes), cervical nodes (distant nodes) and spleens were removed. Cell suspensions were prepared by gentle teasing with 21-gauge needles in medium 199 containing 20% foetal calf serum. Lymphocytes from spleen and heparinized blood were separated from other cell types by a combination of 2 procedures. Phagocytic cells (granulocytes and macrophages) were removed by adherence to a glass-wool column and erythrocytes were separated on a discontinuous density gradient of Ficoll-Hypaque (Yamana, Rolland and Nairn, 1973).

*Cytotoxicity tests* were carried out in Falcon microtitration plates (No. 3034, Falcon Plastics Co.) using a modification of the procedures of Takasugi and Klein (1970). Tumour cell suspension (10 $\mu$l) containing 2·5 or 5·0 $\times 10^4$ cells/ml in medium 199 was placed in each well and allowed to stand at 37°C overnight. The number of cells adherent to the base of each well after washing was then counted by phase contrast microscopy. Wells containing 100–150 cells were used in all tests. Lymphocyte suspension (10 $\mu$l) containing 1·25–2·5 $\times 10^6$ viable cells/ml was then added to each well, giving a final lymphocyte to tumour ratio of 250 : 1. Control lymphocytes were obtained from the corresponding sources in normal, healthy animals. At least 4 replicates of each test well were used. After 4 hours at 37°C, the plates were gently washed twice with warm medium to remove non-adhering lymphocytes and incubated for a further 40–48 hours at 37°C. The plates were then washed twice, fixed and stained with Leishman's stain and the number of tumour cells adhering to each well bottom was counted by light microscopy. Cytotoxicity was expressed as the percentage reduction in the mean number of surviving tumour cells in test (Nt) versus control (Nc) wells, *i.e.*

$$\text{cytotoxicity} = \frac{\text{Nc} - \text{Nt}}{\text{Nc}} \times 100.$$

Student's *t*-tests were performed to estimate the statistical significance of differences between cytotoxicity means and a difference was considered significant at the $P < 0{\cdot}05$ level.

*T and B lymphocytes.*—The relative proportions of T and B cells in the suspensions were determined by membrane immunofluorescence using fluorescein-conjugated anti-rat globulin (Yamana *et al.*, 1973). This conjugate had activity against IgG and IgM and a fluorescein to protein molar ratio of 4·3 : 1. It was absorbed with human liver powder, bovine liver homogenate and washed rat erythrocytes and when used at a globulin concentration of 0·7 g/100 ml gave no significant staining of thymocytes (T cells) but bright membrane staining of a proportion of lymphocytes (B cells) from other lymphoid tissues. A total of 100–200 lymphocytes was counted in each preparation to obtain the percentage of stained cells. Normal values were similarly determined for equivalent tissues from 10 normal, healthy animals.

### RESULTS

*Tumour growth*

Tumours became palpable 2–4 weeks after inoculation (Fig. 1) and then increased rapidly in volume reaching a mean size of 17·1 ± 8·0 $cm^3$ at Week 8. Metastases were observed microscopically in the lungs of some animals at Week 4 and of all animals by Week 6.

Splenomegaly was evident in animals with advanced tumour. Mean spleen weights of tumour bearing animals at Weeks 2 and 8 were 479 ± 43 mg and 728 ±98 mg respectively.

FIG. 1.—Tumour growth curve and evolution of cellular cytotoxicity in lymph nodes. Each point represents the mean of 4 animals. Intermediate node cytotoxicity significantly greater than regional node at Week 6 ($P < 0 \cdot 05$) and Week 8 ($P < 0 \cdot 01$).

FIG. 2.—Evolution of cellular cytotoxicity in regional node, spleen and blood during tumour growth. Each point represents the mean of 4 animals. Spleen and blood cytotoxicity significantly greater than regional node at Weeks 6 and 8. $P < 0 \cdot 001$ (spleen), $P < 0 \cdot 01$ (blood).

*Lymphocytotoxicity*

The time course of cytotoxic reactivity is shown in Fig. 1 and 2, in which mean values are plotted. Cytotoxicity was first detected in regional nodes at Week 2 and in intermediate nodes, spleen and blood at Week 4. After an initial rise, reactivity in regional nodes and blood declined, becoming negligible in regional nodes by Week 6 though intermediate node and spleen reactivities were maintained. Distant nodes showed only a transient response at Week 6. Reactivity of crude spleen suspensions was also assessed but was not found to differ significantly from purified splenic lymphocyte preparations.

TABLE I.—*B Cell Percentages in Various Lymphoid Tissues During Tumour Growth, Mean Values and Standard Error*

| | Lymphocytes | | | | |
|---|---|---|---|---|---|
| | Lymph nodes | | | | |
| Week | Regional | Intermediate | Distant | Spleen | Blood |
| 2 | 19±2·4 | 22±3·3 | 30±1·5 | 32±1·5 | 18±1·5 |
| 4 | 14±2·5 | 15±1·3 | 19±2·1 | 24±2·5 | 20±2·8 |
| 6 | 17±1·2 | 19±1·2 | 24±2·0 | 17±1·5 | 11±1·4 |
| 8 | 20±1·9 | 21±2·1 | 22±1·2 | 20±0·6 | 13±1·5 |
| Normal controls | | 20±0·9 | | 29±3·4 | 16±1·7 |

*T and B lymphocytes*

Relative proportions of T and B cells in the various lymphoid tissues studied are shown in Table I; mean B cell percentages are given. At no time did the results differ significantly from normal values. The apparent rise in blood lymphocyte B cell count at Week 4, the time of maximum blood lymphocyte cytotoxicity, was not found to be statistically significant.

### DISCUSSION

We have observed the development of local lymphocyte anergy in animals with progressively growing tumour. Regional node lymphocytes were at first reactive but later showed no cytotoxicity against the tumour despite persistent anti-tumour reactivity by other lymphoid tissues. The mechanism responsible for the development of this anergy is unclear but the results of the present study show a relationship with the time and extent of tumour growth. Its onset follows the exponential phase of tumour growth, coinciding with the detection of metastases.

Loss of reactivity was not correlated with selective changes in lymphocyte populations as shown by T and B cell proportions. Loring and Schlesinger (1970) have also failed to demonstrate changes in T cell proportions in the lymph nodes of tumour bearing mice, but morphological studies have shown changes in the proportions of immunoblasts and plasma cells in the regional node (Alexander *et al.*, 1969; Edwards *et al.*, 1971).

The observed decline in immunoreactivity of other lymphoid tissues late in tumour growth does not appear to reflect general immunological debilitation as normal responses to sheep erythrocytes and killed *Brucella abortus* organisms have been demonstrated in these animals (Flannery *et al.*, 1973). The response of regional node lymphocytes to phytohaemagglutinin (PHA) was investigated in order to determine whether the anergic state was the result of a general impairment of T cell function. Our results have so far been inconclusive but clinical studies have demonstrated normal (Benezra and Hockman, 1971) and even increased (Fisher, Saffer and Fisher, 1972) response to PHA by regional node lymphocytes of cancer patients.

The source of effector cells responsible for continued activity of spleen and blood lymphocytes is of some interest. Splenomegaly observed in the present study and by others (*e.g.* Blamey and Evans, 1971) suggests the spleen as a possible source (see also Nind *et al.*, 1973).

Serum factors may be responsible for the development of local lymphocyte anergy, acting on tumour (Hellström *et al.*, 1971; Sjögren *et al.*, 1972) or on lymphocytes (Currie and Basham, 1972; Field and Caspary, 1972). Such factors must include some local component, presumably tumour antigen (Alexander, 1970) or antigen–antibody complexes (Baldwin, Price and Robins, 1972; Sjögren

*et al.*, 1972) to account for the early local occurrence of anergy. Blocking activity is currently being investigated and sera from rats of our study, bearing advanced tumours, have been shown to inhibit the lymphocytotoxicity reaction (Flannery *et al.*, 1973).

G.R.F. and P.J.C. are Monash University Graduate Scholars and the work is part of their Ph.D. projects. It is supported by grants from the Anti-Cancer Council of Victoria and the National Health and Medical Research Council. We thank Miss H. Goldsmith and Miss E. Jakimoff for technical assistance.

### REFERENCES

ALEXANDER, P. (1970) Prospects for Immunotherapy of Cancer: Experience in Experimental Systems. *Br. med. J.*, iv, 484.

ALEXANDER, P., BENSTED, J., DELORME, E. J., HALL, J. C. & HODGETT, J. (1969) The Cellular Immune Response to Primary Sarcomata in Rats II. Abnormal Responses of Nodes Draining the Tumour. *Proc. R. Soc. B.*, **174**, 237.

BALDWIN, R. W. (1966) Tumour-specific Immunity Against Spontaneous Rat Tumours. *Int. J. Cancer*, **1**, 257.

BALDWIN, R. W., PRICE, M. R. & ROBINS, R. A. (1972) Blocking of Lymphocyte-mediated Cytotoxicity for Rat Hepatoma Cells by Tumour-specific Antigen–Antibody Complexes. *Nature, New Biol.*, **238**, 185.

BELLONE, C. J. & POLLARD, M. (1970) A Transient Cytotoxic Host Response to the Rous Sarcoma Virus-induced Transplantation Antigen (34851). *Proc. Soc. exp. Biol. Med.*, **134**, 640.

BENEZRA, D. & HOCKMAN, A. (1971) *In Vitro* Activation of Lymphocytes from Patients with Malignant Diseases. I. Kinetics and Differences in Magnitude of Response. *Israel J. med. Sci.*, **7**, 553.

BLAMEY, R. W. & EVANS, D. M. D. (1971) Spleen Weight in Rats During Tumour Growth and in Homograft Rejection. *Br. J. Cancer*, **25**, 527.

CURRIE, G. A. & BASHAM, C. (1972) Serum Mediated Inhibition of the Immunological Reactions of the Patient to His Own Tumour: A Possible Role for Circulating Antigen. *Br. J. Cancer*, **26**, 427.

DISAIA, P. J., RUTLEDGE, F. N., SMITH, J. P. & SINKOVICS, J. G. (1971) Cell-mediated Immune Reaction to Two Gynecologic Malignant Tumors. *Cancer, N.Y.*, **28**, 1129.

EDWARDS, A. J., SUMNER, M. R., ROWLAND, G. F. & HURD, C. M. (1971) Changes in Lymphoreticular Tissue During Growth of a Murine Adenocarcinoma. 1. Histology and Weight of Lymph Nodes, Spleen and Thymus. *J. natn. Cancer Inst.*, **47**, 301.

FIELD, E. J. & CASPARY, E. A. (1972) Lymphocyte Sensitization in Advanced Malignant Disease: A Study of Serum Lymphocyte Depressive Factor. *Br. J. Cancer*, **26**, 164.

FIRKET, H. & LAFONTAINE, N. (1972) Cytotoxicité Spécifique *In Vitro* de Cellules Lymphoides chez des Souris Porteuses d'une Greffe Cancéreuse. Evolution au Cours de la Croissance de la Tumeur. *C.r. Acad. Sci. Paris*, t.275, Série D, 1579.

FISHER, B., SAFFER, E. A. & FISHER, E. R. (1972) Studies Concerning the Regional Lymph Node in Cancer. III. Response of Regional Lymph Node Cells from Breast and Colon Cancer Patients to PHA Stimulation. *Cancer, N.Y.*, **30**, 1202.

FLANNERY, G. R., CHALMERS, P. J., ROLLAND, J. M. & NAIRN, R. C. (1973) Immune Response to a Syngeneic Rat Tumour: Evolution of Serum Cytotoxicity and Blockade. *Br. J. Cancer*, **28**. In the press.

HELLSTRÖM, K. E. & HELLSTRÖM, I. (1971) Some Aspects of the Immune Defense Against Cancer. I. *In Vitro* Studies on Animal Tumors. *Cancer, N.Y.*, **28**, 1266.

HELLSTRÖM, I., SJÖGREN, H. O., WARNER, G. A. & HELLSTRÖM, K. E. (1971) Blocking of Cell-mediated Tumor Immunity by Sera from Patients with Growing Neoplasms. *Int. J. Cancer*, **7**, 226.

DE LANDAZURI, M. O. & HERBERMAN, R. B. (1972) Immune Response to Gross Virus-induced Lymphoma. III. Characteristics of the Cellular Immune Response. *J. natn. Cancer Inst.*, **49**, 147.

LORING, M. & SCHLESINGER, M. (1970) The Θ Antigenicity of Lymphoid Organs of Mice Bearing the Ehrlich Ascites Tumor. *Cancer Res.*, **30**, 2204.

NAIRN, R. C. (1973) The Immunology of Malignant Melanoma. In *Immunology of Skin Diseases*. Ed. L. Fry and P. P. Seah. Oxford: M.T.P. In the press.

NAIRN, R. C., NIND, A. P. P., GULI, E. P. G., DAVIES, D. J., ROLLAND, J. M., MCGIVEN, A. R. & HUGHES, E. S. R. (1971) Immunological Reactivity in Patients with Carcinoma of Colon. *Br. med. J.*, iv, 706.

NAIRN, R. C., NIND, A. P. P., GULI, E. P. G., DAVIES, D. J., LITTLE, J. H., DAVIS, N. C. & WHITEHEAD, R. H. (1972) Anti-tumour Immunoreactivity in Patients with Malignant Melanoma. *Med. J. Aust.*, **1**, 397.

NIND, A. P. P., NAIRN, R. C., ROLLAND, J. M, GULI, E. P. G. & HUGHES, E. S. R. (1973) Lymphocyte Anergy in Patients with Carcinoma. *Br. J. Cancer*, **28**, 108.

OETTGEN, H. F., OLD, L. J. & BOYSE, E. A. (1971) Human Tumor Immunology. *Med. Clins N. Am.*, **55**, 761.

SJÖGREN, H. O., HELLSTRÖM, I., BANSAL, S. C., WARNER, G. A. & HELLSTRÖM, K. E. (1972) Elution of " Blocking Factors " from Human Tumors Capable of Abrogating Tumor-cell Destruction by Specifically Immune Lymphocytes. *Int. J. Cancer*, **9**, 274.

TAKASUGI, M. & KLEIN, E. (1970) A Microassay for Cell-mediated Immunity. *Transplantation*, **9**, 219.

VÁNKY, F. & STJERNSWÄRD, J. (1971) Tumor-distinctive Cellular Immunity to Human Sarcoma and Carcinoma. *Israel J. med. Sci.*, **7**, 211.

YAMANA, S., ROLLAND, J. M. & NAIRN, R. C. (1973) T and B Cells in Various Lymphoid Tissues in Mice. *Immunol. Commun.* In the press.